# Author Correction: Exploring the space of self-reproducing ribozymes using generative models

**DOI:** 10.1038/s41467-025-67592-w

**Published:** 2025-12-18

**Authors:** Camille N. Lambert, Vaitea Opuu, Francesco Calvanese, Polina Pavlinova, Francesco Zamponi, Eric J. Hayden, Martin Weigt, Matteo Smerlak, Philippe Nghe

**Affiliations:** 1 Laboratoire de Biophysique et Evolution, UMR CNRS-ESPCI 8231 Chimie Biologie Innovation, ESPCI Paris, Université PSL, Paris, France; 2 Max Planck Institute for Mathematics in the Sciences, Leipzig, Germany; 3 Sorbonne Université, CNRS, Laboratoire de Biologie Computationnelle, Quantitative et Synthétique, UMR 7238, Paris, France; 4 Dipartimento di Fisica, Sapienza Università di Roma, Rome, Italy; 5 Biomolecular Sciences Graduate Programs, Boise State University, Boise, ID USA; 6 Department of Biological Sciences, Boise State University, Boise, ID USA; 7 Capital Fund Management, Paris, France

**Keywords:** Origin of life, High-throughput screening

Correction to: *Nature Communications* 10.1038/s41467-025-63151-5, published online 22 August 2025

In the version of the article initially published, in the “Construction of the MSA” section of the Methods, the text “the PDB X-ray structure 1G9B” should have read “the PDB X-ray structure 1U6B” and has now been corrected in the HTML and PDF versions of the article.

