# Supplementary material for: Exploring the space of self-reproducing ribozymes using generative models
Source: Nat Commun. 2025 Aug 22;16:7836. doi: 10.1038/s41467-025-63151-5 (PMC12373953; doi:10.1038/s41467-025-63151-5)
Supplement: Supplementary file 1 — Supplementary Information [file 41467_2025_63151_MOESM1_ESM.pdf]

Supplementary Information of

Exploring the space of self-reproducing ribozymes using generative models

by Lambert, Opuu, Calvanese et al.

Models

Profile (PRO)

Each position is drawn independently from the distribution of nucleotides at the corresponding position in the MSA. In this case, the frequencies are computed while omitting gaps to sample full sequences. The distribution of nucleotides per position is shown in Supplementary Figure 11.

Base-Pair Replacement (BPR)

The complementary design strategy consists in sampling only sequences that are compatible with the known secondary structure of the Azoarcus ribozyme. Note that this is only a compatibility condition, which does not guarantee that the minimum free energy structure is indeed the Azoarcus ribozyme one. In practice, for paired positions in the structure, including the pseudoknot, we sampled pairs of nucleotides that can form canonical base pairs (A, U), (C, G), and (G, U). For the unpaired position, nucleotides were drawn uniformly. Therefore, sequences sampled with this strategy are compatible with the Azoarcus ribozyme structure, which however does not mean the minimum free energy structure is that of the Azoarcus ribozyme.

Direct Coupling Analysis (DCA)

In Direct Coupling Analysis, each nucleotide sequence is described by a Boltzmann-like probabilistic model: $P(n1 \dots nL) \propto exp(-H(n1, \dots nL))$, where the Hamiltonian $H(n1, \dots nL) = \sum h_i(a) + \sum J_{ij}(a,b)$ represents the log-likelihood. Here, high probability sequences are sequences that restitute best the statistical signature of natural counterparts.

The training of the DCA T=1 model was conducted using the methods and code provided in reference Calvanese et al.[1]. Sequences were sampled from the model at fixed distances from the Azoarcus reference sequence, with distance values ranging from 5 to 90 in increments of 5. For each distance value, 150 sequences were sampled using Gibbs sampling, ensuring equilibrium was reached. The generation was biased toward the reference sequence using a biasing potential $H(n1, \dots, nL) = \sum hi(a) + \sum Jij(a,b) + \theta \cdot distance(a, azo)$. For each distance value, the 150 sequences were randomly selected from those below the 10% quantile of the corresponding distance DCA energy (10% best DCA score). The training of the DCA model for DCA T=0.3 and DCA+SB was conducted using the procedure described in Cuturello et al.[2]. To determine the parameter h_i and J_{ij}, we started with the observed frequencies $f(n,i)$ of nucleotide n at each position i in the MSA.

The parameters were updated in order to reproduce these single and pairwise frequencies (Supplementary Figures 11 and 12). For the learning procedure, we used N=20 × 10^3 MC sweeps, K=20 × 10^3 MC steps. MC sweeps were run at kT=0.3 with a regularization λ = 0.3. Once the model trained, we compared the predicted frequencies f_i, f_{ij} to the ones observed in MSA and obtained Pearson's correlation of $\rho = 0.99$ for f_{ij}, and $\rho = 0.96$ for connected correlations $c_{ij}(a,b) = f_{ij}(a,b) - f_i(a) \times f_j(b)$, the latter being a consistency check as they are not directly fitted by the procedure (Supplementary Fig. 12). This update has been done using a non-persistent contrastive divergence algorithm as described in Cuturello et al.[2] (see pseudo-code in Supplementary Fig. 13).

We generated sequences by sampling first k positions to be mutated, which were drawn randomly from 10 to 100 by step of 10 mutations. For each k positions selected, we performed 100 sweeps of $k \times 200$ MCMC steps used in the metropolis test with the DCA energy parametrized above. Then, we pooled all the designed sequences and populated bins of 10 (from 0-10 to 80-90) with the 280 sequences with the best DCA score and at least 5 mutations from each other.

Variational AutoEncoder (VAE)

For the variational autoencoder (VAE), we derived our implementation from reference Ding et al.[3]. A VAE is composed of three main elements: an encoder neural network, a decoder neural network, and a latent space. The encoder (denoted He) is used to find the underlying data structure of the MSA by projecting sequences into a low dimensional latent space Z. The main difference of this latent space is that Z is modeled as a Gaussian variable.

To generate sequences, the decoder network is used to convert latent representation into sequences. To train this model, we used the evidence lower bound (ELBO) loss, which composed of two terms:

$$ELBO(\theta, \phi) = \sum_{Z} q_{\phi}(Z \vee X) log p_{\phi}(X \vee Z) - \sum_{Z} q_{\phi}(Z \vee X) log \frac{q_{\phi}(Z \vee X)}{p_{\phi}(Z)}$$

where the θ are the weights of the encoder and the φ are the ones for the decoder. For each sequence, this loss evaluates first the log-likelihood predicted by the decoder network (the first term), then compute the divergence of the Z representation produced by the encoder from its prior Gaussian distribution (the second term).

To choose the best architecture, we tried several hyper parameters with a 5-fold cross-validation procedure. We selected the architecture with two layers of 512 hidden units (for both encoder and decoder), and 128 dimensions for the latent space, with a ReLU activation function. The decoder predicts log-probabilities that are converted back using the Softmax function over the four nucleotides. To read the predicted sequence from the output of the decoder, we chose at each position the nucleotide with the highest probability.

To sample artificial RNA sequences, we drew randomly 60000 data points in the latent space using a Gaussian distribution centered at the coordinate of the wild type using several variances $\sigma \in 0.1,0.9,1,10$. Then, we decoded all latent data points into sequences. Then, we populated bins of 10 with 280 randomly selected sequences, from 1 to 100 mutations, where at least five mutations are observed between each pair of designs.

Structure-Based (SB)

To predict the secondary structure of a sequence, we used the thermodynamic energy model called nearest neighbor with the Turner2004 parameters from Mathews et al.[4]. Here, the parameters give the free energy of folding of a sequence into a given structure, where the associated probability is $p(s) = exp\{-\Delta G(s)\}$. The Zuker algorithm[5], a dynamic programming algorithm, allowed us to compute the minimum free energy structure of a given sequence for this energy model. However, this algorithm does not account for pseudoknots.

To measure the selectivity of one sequence to adopt a specific secondary structure among all possible secondary structures, we used McCaskill's algorithm[6], which is a variant of Zucker's algorithm. This algorithm enumerates all possible secondary structures, in contrast with the prediction of the minimum free energy structure, and records the probability of each pair of positions to be paired in this ensemble. The probabilities of pairing are reported into a matrix called base pair probability matrix (BPPM). We obtained the BPPM using the implementation of McCaskill's algorithm in the ViennaRNA package (version 2.5.17) (Supplementary Fig. 6e).

Similarly, to Zuker's algorithm, this does not account for pseudoknots; however, we noticed residual probabilities at positions involved in the pseudoknot, as shown in Supplementary Fig. 6e. We compared the BPPM predicted by ViennaRNA[7], and the algorithm of NUPACK[8] that explicitly accounts for pseudoknots (Supplementary Fig. 6d). However, the prediction with accounting explicitly for pseudoknots does not yield a drastic improvement while being one order of magnitude slower.

The structure recovery score (SB) is based on the difference between the predicted BPPM and the known secondary structure of Azoarcus GII, where $\delta_{ij\in\sigma}$ is 1 if positions ij are paired in the known structure (denoted σ here). $BPPM_{i,j}$ is the predicted probability of i, j are paired for the sequence s.

Tertiary structure constraints (3D)

To favor the recovery of tertiary interactions and the catalytic core, we devised additional constraints where we fixed the wild type nucleotide of Azoarcus at positions involved in the IGS, the terminal G, the tetraloop motif (GAAA), the P7-loop involved in the catalytic core, and positions involved in tertiary interactions as delineated in Mustoe et al.[9] using an X-ray structure and molecular dynamics simulation. In total, 66 out of 197 positions were held fixed during the sequence space exploration, as shown in Supplementary Fig. 6a. We imposed these constraints in the context of the BPR model and the SB model.

Direct Coupling Analysis & Secondary structure (DCA-SB)

To generate sequences from DCA and the secondary structure score, we combined both scores in the metropolis test of the MCMC with an acceptance score $exp([\alpha DCA(s) + SB(s)]/T)$. To sample sequences, we performed the same protocol as for the DCA alone generated sequences, considering several α values: $0.1, 0.3, 0.4, 0.7$. The goal was to reduce the importance of the DCA contribution, enabling the exploration of sequence driven by the biophysical model. Once the sequences were sampled, we pooled all the designs and populated bins of 10 mutations (from 0-10 to 90-100 mutations) with the 280 sequences having the best DCA score, but with at least 5 mutations from each other.

Benchmarking on experimental data

We benchmarked the DCA scores and the SB score on experimentally generated data published earlier[10]{Citation}. In this study, the authors started with the wild type sequence of Azoarcus GII, and synthesized a pool of variants where five positions were randomly mutated. To ensure sufficient diversity, they also randomly introduced additional mutations using PCR prone mutagenesis. Using this protocol to generate sequence diversity, they designed four pools of many thousand RNA sequences, representing four strengths of selection pressure for the catalytic activity. To control the strength of the selection pressure, the authors used the concentration of MgCl2 (mM), allowing them to discriminate the very active variants that were able to perform the selected catalytic activity with low MgCl2 ('Str' at [MgCl2] = 2 nM), see Supplementary Table 4. In contrast, poorly active variants only appeared in the non-selected batch ('Pre').

To test our scoring functions (DCA and SB), we first aligned each sequence to the wild type one, using the global alignment algorithm that only accounts for matches and mismatches. Because of the experimental protocol used in this reference, 20 positions were not mutated. Supplementary Figure 14 shows the distribution of DCA scores (relative to the wild type, which is set to zero) per pool of mutants, where lower is better. As expected, the distribution is ordered by the strength of the selection pressure which has been used; therefore, showing that the DCA score indeed captures to some extent the catalysis of GII. Moreover, the lowest DCA score here is zero (the wild type), consistent with the assumption that natural GII are more efficient than artificial mutants. Similarly, in Supplementary Figure 14, we performed the same calculation with the SB score, which also yielded comparable results. We compared the SB score with the ensemble defect (ED)[11] where both performed similarly. We chose the SB score because it displayed a larger difference between the *Pre* (no selection) and *Str* (strongest selection) pools.

Contact predictions

To predict the contacts between pairs of positions using H^{azo}, we computed the Average Product Correction (APC) F_{ij}^{APC} for each pair of positions. For this system, we used the 1% highest F_{ij}^{APC} (188 pairs of positions) as prediction of contacts (Supplementary Fig. 6c). We compared the prediction with the X-ray structure (contacts are defined by a cutoff of 3.5Å on the minimum distance between nucleotides heavy atoms). 54.7 % of the predictions are correct according to the secondary structure or the considered X-ray structure, while 83% of the secondary structure contacts were correctly predicted. Moreover, some tertiary contacts were also recovered (dark circles, Supplementary Fig. 6c).

Phormidium

To confirm our results, we designed sequences starting not from Azoarcus wild type GII but Phormidium GII—a 208 nucleotide long sequence. For Phormidium, we built the MSA based on the 2611 sequences of GII in RFAM (RF00028), where we selected 1424 sequences (compared to the 815 selected for Azoarcus), which we then aligned. To search and align Phormidium homologs, we used Infernal with a seed constituted only with Phormidium wild type sequence obtained from the database GISSD and a secondary structure obtained by Shape-Map experiment (Supplementary Fig. 9a).

From the DCA model, we designed 1341 sequences across 10 bins of 10 mutations (0-10, 10-20, …) populated with roughly 140 sequences each. Similarly, with Azoarcus, we combined

DCA with the SB score, where the DCA score is scaled with α = 0.5. To sample sequences with mutations ranging from 0 to 90, we used seven MCMC sweeps of $2.1 \cdot 10^6$ steps with a biasing potential based on the distance to the wild type to drive the MCMC toward more or less mutated sequences. Supplementary Fig. 9b shows the activity measured by deep sequencing across the different bins of number mutations. These results support our findings with Azoarcus: i) Azoarcus is not a special point as our design exploration also works with another GII, and ii) the SB score improved the exploration of the sequence space by a similar amount. DCA alone displayed an L_{50}=25, whereas with the contribution of secondary structure yielded an L_{50}=35.

Support size computations

In a strict sense, the support X of a probability distribution $P(x)$ is the set of all outcomes x that have a non-zero probability, and the support size is the cardinal Ω of X. The *effective* support size is an estimation of the number of different outcomes you can expect in practice when sampling from the distribution. For instance, with a fair dice, you expect 6 different outcomes. However, for a rigged dice where the face displaying '6' has a probability of $\frac{1}{1000}$ instead of $\frac{1}{6}$, you would expect an effective support size closer to 5 because this face is rarely seen. One measure of the effective support is the exponential of the entropy of the probability distribution $S = -\sum_{x \in X} P(x) log\ P(x)$. In the dice example, the effective support size of the fair dice computed from entropy equals $2^{2.58\ldots} = 6$ and equals the actual support size. For the rigged dice the effective support size is $2^{2.33\ldots} = 5.03$, consistent with our intuitive expectation. This choice is justified from results of the book of Cover and Thomas *Elements of Information Theory* cited in the main text, summarized below.

For models such as Random Uniform Mutagenesis, Random Base Pairs, Random Base Pairs with 3D constraints, the distribution $P(x) = \frac{1}{\Omega}$ is equiprobable over Ω states, and

$$S = -\sum_{x \in X} P(x) log\ P(x) = \sum_{x \in X} \frac{1}{\Omega} log\ \frac{1}{\Omega} = log\ \Omega.$$

As in the fair dice example, the actual support size Ω of uniform probability distributions coincides with the effective support size computed from the entropy as $exp\ (S) = \Omega$.

In the non-equiprobable cases (Profile, SB, DCA, DCA-SB), consider N independent samples $x^i \in X$ from a distribution $P(x)$ with entropy S. For any $\varepsilon > 0$, one defines the *typical set* $A_\varepsilon^{(N)} \subset X^N$ as the set of N-tuples verifying:

$$e^{-N(S+\varepsilon)} \le P(x^1, x^2, \ldots, x^N) \le e^{-N(S-\varepsilon)}.$$

Theorem 3.1.2 of Cover & Thomas[12] (p. 59) states that:

$$Pr\left(A_\varepsilon^{(N)}\right) \ge 1 - \varepsilon$$

and

$$(1 - \varepsilon) e^{N(S-\varepsilon)} \le \left|A_\varepsilon^{(N)}\right| \le e^{N(S+\varepsilon)}$$

where Pr is the probability distribution over N-tuples, for arbitrarily small ε provided that N is sufficiently large. Colloquially, this means that, for N large, almost all samples of size N are part of a typical set whose size is $|A_\varepsilon^{(N)}| \approx exp(S)^N$.

Instead of considering the most typical sets, Theorem 3.3.1 (p. 63) considers the most probable set $B_\delta^{(N)}$, which comprises the most probable N-tuples such that $Pr\left(B_\delta^{(N)}\right) \geq 1 - \delta$, with δ arbitrarily small. The theorem states that

$$|B_\delta^{(N)}| > e^{N(S-\eta)}$$

where both δ and η may be arbitrarily small provided that N is sufficiently large. Colloquially, this means that any arbitrarily large fraction of the most frequent N-tuples comprises at least $exp(S)^N$ elements, for N large enough.

Taken together, these two theorems consistently show that the probability of N-tuples becomes increasingly uniform and behaves like $\left(\frac{1}{\Omega}\right)^N$ with $\Omega = exp(S)$ as N increases (formula 3.27, p. 63, Cover and Thomas). In other words, N-tuple probabilities behave as if one were sampling N times from a uniform distribution of size $exp(S)$. A possible way to understand this result is that, when probabilities of single draws are uneven, in long series, it becomes exponentially less likely to draw an element outside of a certain set of size Ω as compared to resampling an element that has already been seen because it is more probable.

The same notions admit an interpretation in thermodynamics, which we briefly present as it may help readers familiar with statistical physics, following Kardar[13] (pp. 110-113). Consider a system described by the Boltzmann probability distribution:

$$P(x) \sim exp\,\{-H(x)\}$$

The microstates are no longer equiprobable; their probability is determined by the energy function $H(x)$ (in our case, Profile, SB, DCA, and DCA-SB correspond to this case). Note that all the system microstates with the same energy have the same assigned probability. The energy function is extensive, meaning that it scales linearly with the size of the system L (the sequence length for an RNA system), i.e.,

$$H(x) = L \cdot e(x)$$

With $e(x)$ being the energy density. In the thermodynamic limit (i.e., $L \to \infty$) there exists an energy density value e_{avg} around which the state probability distribution becomes sharply peaked. Thus, drawing from the probability distribution yields a state with energy density close to e_{avg} with probability tending to 1. The distribution entropy S in this case corresponds to

$$S \approx log\,\Omega\left(e_{avg}\right)$$

Since a finite sample will never exhibit extensive energy differences (or finite differences in energy density), the sampled states, with probability close to 1, will belong to the set of $\Omega(e_{avg}) = exp\,(S)$ states around e_{avg}.

Below, for some models, we could compute the support size exactly from combinatorics, in other cases we computed approximate estimations, at any given mutational distance K. However, in some cases, such as the VAE model, there is no known strategy to compute the effective support size.

Random Uniform Mutagenesis

Calculating the support size for random uniform mutations consists of determining how many possible mutations exist for the wild type sequence a given distance K. To do this, we count in how many different ways we can choose K sites out of 193 and consider that each site can mutate in 3 different ways (since there are 3 possible mutations per site if we exclude the original base).

$$\Omega_K = \binom{193}{K} \cdot 3^K$$

Base-Pair Replacement

To compute the support size for the secondary structure-compatible random mutations, consider the 59 Watson-Crick contacts (including those involved in the pseudoknot) and 75 'free' residues not engaged in Watson-Crick pairing. Each of the 75 free residues can mutate in 3 different ways. All the Watson-Crick pairs can mutate in 5 different ways (considering also the wobble pair GU). The mutational possibilities for the wild type pairs are diagrammed as follows:

Original pair	2 mutations from the wild type	1 mutation from the wild type
AU	UA, CG, GC, UG	GU
GC	UA CG, AU, UG	GU
GU	UA, CG, UG	GC, AU

Except for the wobble pairs GU, a base pair can undergo 4 types of mutations when both residues mutate, and 1 type of mutation if only one residue mutates. Since only 6/59 pairs are GU in the wild type, the calculation has been done assuming the behavior of Watson Crick pairs for all secondary-structure pairs.

We define z as the number of Watson-Crick pairs mutating by introducing one mutation relative to the wild type, y as the number of pairs introducing two mutations from the wt, and x as the number of unpaired sites mutating. The combinatorial size for K mutations can be expressed as:

$$\Omega_K = \sum_{z=1}^{59} \binom{59}{z} \sum_{x+2y=K-z} \binom{75}{x} \cdot 3^x \cdot \binom{59-z}{y} \cdot 4^y$$

where the second sum is performed over the solution of the equation considering that

$$x \in \{0, \cdots, 75\}$$
$$y \in \{0, \cdots, 59-z\}$$

For any given z in the first sum, the condition on the second sum ensures that we reach exactly K mutations from the wild type.

Base-Pair Replacement 3D

The calculation of the support size for the BPR-3D is exactly the same as for the BPR, except that 17 Watson-Crick pairs and 28 free residues are kept fixed. We are left with 59-17 = 42 Watson-Crick pairs and 75-28 = 47 free residues

$$\Omega_K = \sum_{z=1}^{42} \binom{42}{z} \sum_{x+2y=K-z} \binom{47}{x} \cdot 3^x \cdot \binom{42-z}{y} \cdot 4^y$$

with:

$$x \in \{0, \cdots, 47\}$$
$$y \in \{0, \cdots, 42 - z\}.$$

Energy based models

For the energy-based models such as Profile, DCA T=1, covariance, DCA+SB, and DCA T=0.3, each outcome at a given distance is not equiprobable, so we need to estimate the effective support size as the exponential of the Shannon entropy.

The energy function $H(n)$ defines a probability distribution over the sequence space, $P(n) = \frac{1}{Z} exp(-H(n))$. To estimate the entropy of $P(n|K)$, we use Bayes' theorem, which connects these two probabilities. Indicating with B_K the set of sequences at distance K from the wild type we have:

$$P(n|K) = \frac{P(n,K)}{P(K)} = \frac{P(n) \cdot \delta\{K, dist(n,wt)\}}{P(K)} = \frac{1}{Z} \frac{exp\{-H(n)\} \cdot \delta\{K, dist(n,wt)\}}{P(K)}$$
$$S_K = log(Z) + [H(n)]_{B_K} + log(P(K))$$

where $P(K)$ is the probability that a generated sequence is at distance K from the wildtype (wt). Analytically it is the sum of $P(n)$ over all sequences at that distance,

$$P(K) = \frac{1}{Z} \sum_{n \in B_K} exp\{-H(n)\}$$

but it can be computed by generating a number of sequences and counting how many of them are at distance K. The partition function Z is unknown and requires a summation over the entire sequence space, which is impossible to perform in practice.

Adding a biasing potential to the model $H_\theta(n) = H(n) + \theta \cdot dist(n,wt)$, as done for the sampling of DCA T=1 designs, does not change $P(n|K)$ or S_K:

$$P_\theta(n|K) = \frac{\frac{1}{Z_\theta} \exp\{-H_\theta(n)\} \cdot \delta\{K, dist(n,wt)\}}{\frac{1}{Z_\theta} \sum_{n \in B_K} \exp\{-H_\theta(n)\}} = \frac{\frac{1}{Z} \exp\{-H(n)\} \cdot \delta\{K, dist(n,wt)\}}{\frac{1}{Z} \sum_{n \in B_K} \exp\{-H(n)\}} = P(n|K)$$

We use this property, along with the fact that $S_0 = 0$ (the mutational space at 0 mutations only has the wild type, so the probability distribution $P(n|0)$ has only one possible outcome and an entropy $S_0 = 0$) to compute S_K for all K of interest.

We take a series of biasing potentials $\{\theta_1, \theta_2, \ldots, \theta_N\}$ such that θ_1 provides good coverage of the mutational distances in $\{0, \ldots, K_1\}$ (meaning that sampling from $H(n) + \theta_1 \cdot dist(n,wt)$ we generate enough sequences with the specified mutational distances to allow for statistical analysis). Similarly, θ_2 ensures good coverage from $\{K_1, \ldots, K_2\}$, and in general, θ_i provides good coverage in $\{K_{i-1}, \ldots, K_i\}$.

The biasing potential θ_1 is strong enough that some of the generated sequences will be at distance 0 from the wildtype. Using the entropy formula at distance 0, we obtain $log(Z_{\theta_1})$

$$S_0 = log(Z_{\theta_1}) + [H_{\theta_1}(n)]_{B_0} + log(P_{\theta_1}(K=0))$$
$$log(Z_{\theta_1}) = -log(P_{\theta_1}(K=0)) - [H_{\theta_1}(n)]_{B_0}$$

Having the value of $log(Z_{\theta_1})$, we can compute S_K for all distances $\{0,\dots,K_1\}$ for which we have a large enough sample to reliably estimate $P_{\theta_1}(K)$ and H_{θ_1}. We then relax the bias to θ_2 to increase the distance from the wt. To compute $log(Z_{\theta_2})$, we cannot again use the $S_0 = 0$ trick since the less biased model $H_{\theta_2}(n)$ will not generate enough sequences at distance 0 from the wt. However, we know that there is K^1 with good overlap between the θ_1-biased model and the θ_2-biased model, so we can compute S_{K^1} from the θ_1 model and then use it to compute $log(Z_{\theta_2})$.

$$S_{K^1} = log(Z_{\theta_1}) + [H_{\theta_1}(n)]_{B_{K^1}} + log(P_{\theta_1}(K^1))$$
$$S_{K^1} = log(Z_{\theta_2}) + [H_{\theta_2}(n)]_{B_{K^1}} + log(P_{\theta_2}(K^1))$$
$$log(Z_{\theta_2}) = S_{K^1} - log(P_{\theta_2}(K^1)) - [H_{\theta_2}(n)]_{B_{K^1}}$$

This procedure can be iterated to compute $log(Z_i)$ for all the biased models and S_K for all desired distances, considering that:

$$S_{K^{i-1}} = log(Z_{\theta_{i-1}}) + [H_{\theta_{i-1}}(n)]_{B_{K^{i-1}}} + log(P_{\theta_{i-1}}(K^{i-1}))$$
$$S_{K^{i-1}} = log(Z_{\theta_i}) + [H_{\theta_i}(n)]_{B_{K^{i-1}}} + log(P_{\theta_i}(K^{i-1}))$$
$$log(Z_{\theta_i}) = S_{K^{i-1}} - log(P_{\theta_i}(K^{i-1})) - [H_{\theta_i}(n)]_{B_{K^{i-1}}}$$

As observed in Supplementary Table 2, for DCA T=0.3 and DCA-SB the support size estimation was not possible after a certain number of mutations due to ergodicity problems. This means that the Markov Chain Monte Carlo (MCMC) simulations do not fully thermalize and we cannot estimate equilibrium properties like the entropy. However, this does not hinder sequence generation since the chains, despite not being thermalized, still explore regions with favorable model scores (low energy). DCA T=1 support sizes have been reduced by a factor of ten because we only experimentally tested the sequences in the top 10% quantile of the DCA score. This approach is conservative since it assumes that the remaining 90% of sequences are non-active.

Supplementary Figures

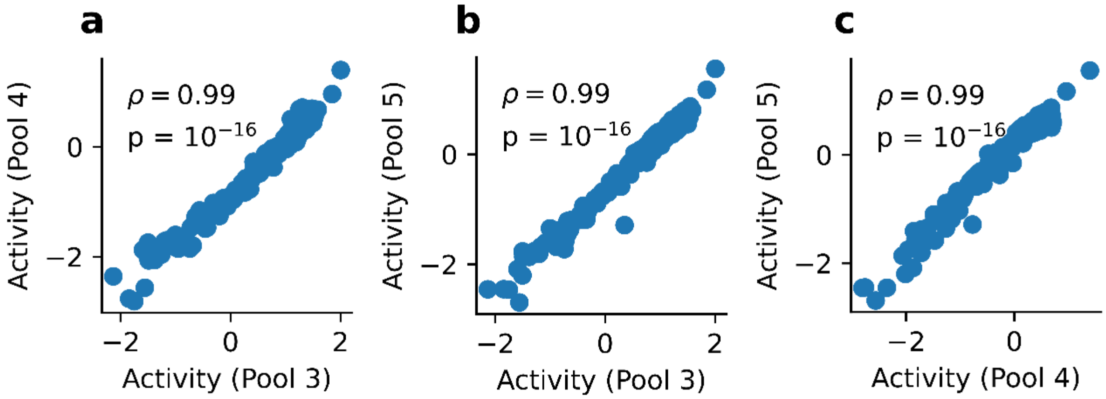

Supplementary Figure 1. Assay reproducibility. We compared the activity computed for the same set of sequences from 3 independent experiments called Pool 3, Pool 4, and Pool 5. The Pearson correlation ρ for each comparison is 0.99 for N=355 with two-sided $p=10^{-16}$, which is the numerical precision.

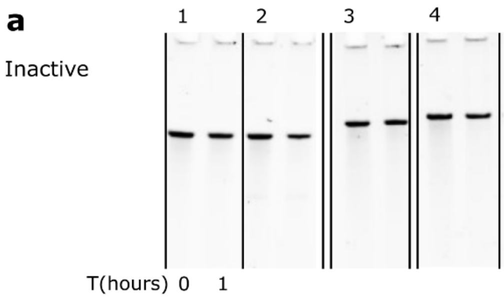

Column	Sequence Name	Step 2	Step 1+2
1	dca.T1.30.1	-2.275	-4.698
2	dca.T1.45.1	-1.964	-4.687
3	dca.T1.60.3	-1.450	-3.046
4	dca.T1.45.2	-1.804	-3.440

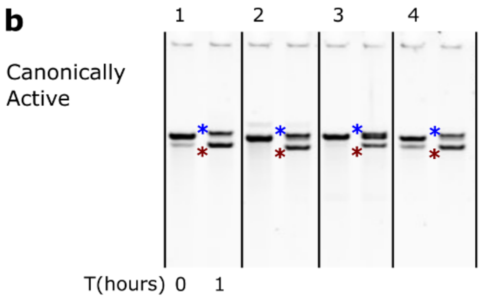

Column	Sequence Name	Step 2	Step 1+2
1	dca.T1.15.4	0.511	0.479
2	dca.T1.30.5	-0.375	-0.397
3	dca.T1.45.5	-0.321	-0.357
4	Azo	0	0

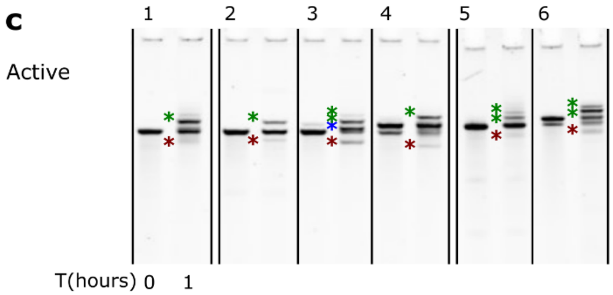

Column	Sequence Name	Step 2	Step 1+2
1	dca.T1.15.3	-0.156	-1.059
2	dca.T1.45.3	-0.788	-2.168
3	dca.T1.30.4	0.215	-1.499
4	dca.T1.45.4	-0.075	-1.168
5	dca.T1.55.1	-0.358	-1.522
6	dca.T1.60.1	0.570	-1.232

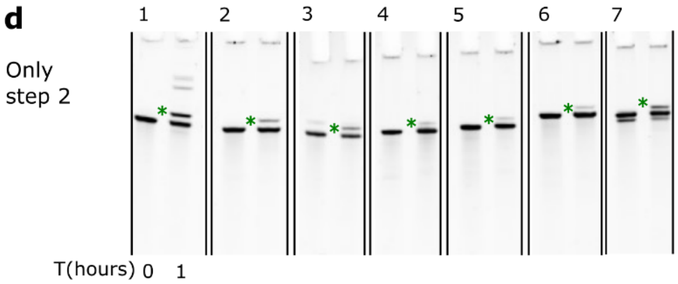

Column	Sequence Name	Step 2	Step 1+2
1	dca.T1.15.1	-0.164	-4.047
2	dca.T1.30.3	-0.713	-2.498
3	dca.T1.45.6	-0.599	-1.365
4	dca.T1.50.1	-0.612	-1.293
5	dca.T1.50.2	-1.110	-1.654
6	dca.T1.60.4	-1.109	-2.870
7	dca.T1.60.2	0.007	-1.330

Supplementary Figure 2. Gel electrophoresis analysis of designed sequences. Left: Denaturing gel electrophoresis of self-splicing assay, grouped by category. Red stars indicate products of step 1, blue stars products of step 1 followed by step 2, green stars products of step 2 without step 1. Inactive: only the candidate ribozyme is visible, none of the products are visible. Canonically active: products of step 1 and step 2 are visible, such that step 1 mostly occurs before step 2. Active: products of step 1 and step 2 are visible, but step 2 may happen while step 1 has not yet occurred. Only step 2: only products of step 2 are visible. Right: scores as measured in the pooled sequencing assay, for step 2 only and steps 1 followed by step 2 (denoted step 1+2).

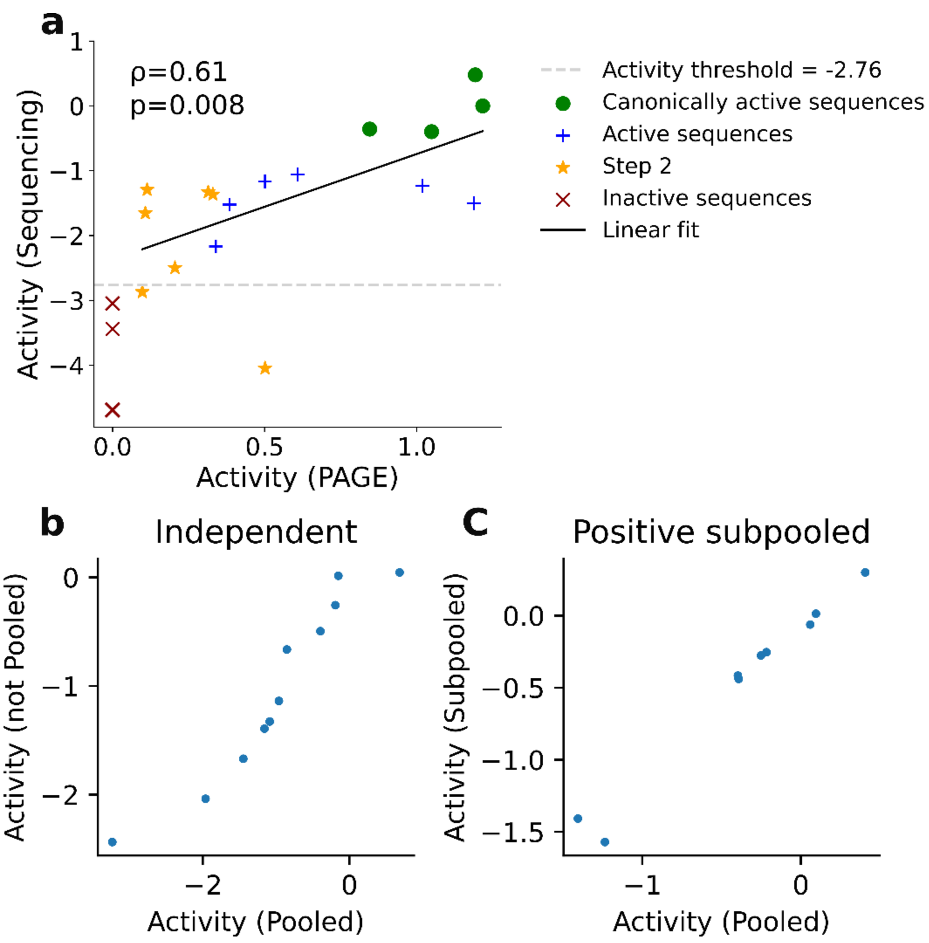

Supplementary Figure 3. Cross-catalysis tests. a, Comparison of the catalytic activity of the 20 mutants of Supplementary Figure 2, measured separately and by two independent methods: PAGE (x-axis) and sequencing assay. Categories as represented by different types of dots correspond to those described in Supplementary Figure 2. Inactive sequences (red crosses) do not display any visible bands on PAGE (thus have a x-value of 0) and consistently, their sequencing score is below the sequencing activity threshold (dotted line). The PAGE activity of all other mutants is reported as the total activity (step 1 + step 2 of the assay), obtained by integrating the band intensities distinct from the band corresponding to the sequence before reaction, and normalizing by the initial band intensity. The 2 activity scores restricted to active sequences follow an affine relationship (affine least square regression as indicated within the figure), and correlate significantly with a Pearson correlation of 0.61 (two-sided p=0.008, N=17, yellow dot outlier included). **b-c,** Tests of cross-catalysis. Comparison of sequencing-based scores: **b,** For a subset of sequences spanning a range of activity scores, as measured within a large pool of 18 000 sequences (x-axis) versus measured independently from each other; **c,** Subset sequences found to be positive in the large pool assay (x-axis, together with 18.000 sequences), versus activity score measured when incubated as a subpool.

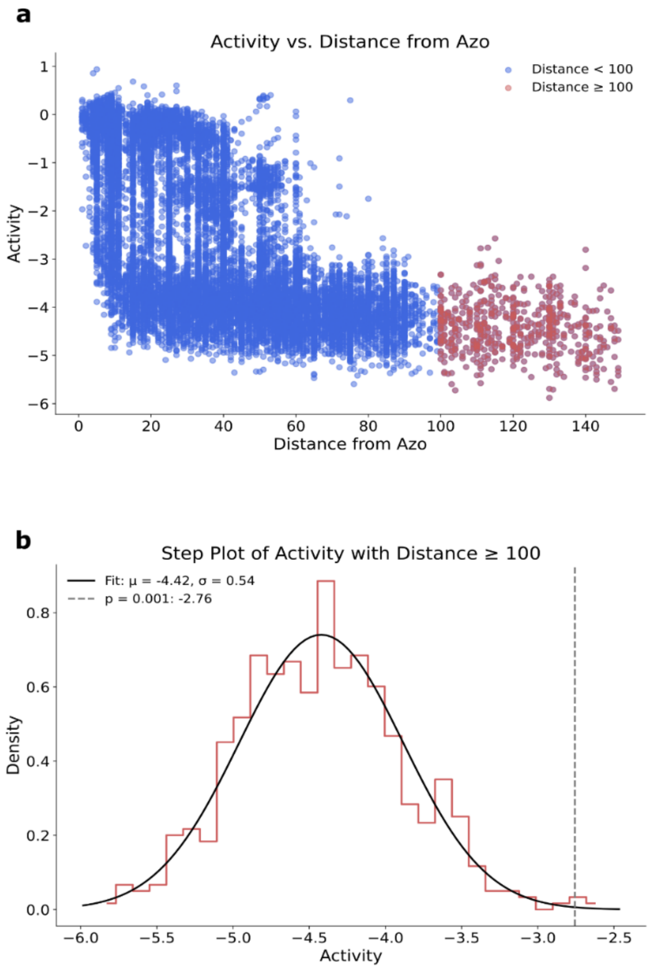

Supplementary Figure 4. Activity score noise distribution. a, Scatter plot of the experimental activity against the distance from the wild type for the 15147 sequences that exhibited experimental activity ($f_{sel} > 0$). In red, the 543 designs that introduce 100 or more mutations on the Azo reference. Their activity values are considered representative of experimental noise. **b,** Gaussian fit on the activities of the 543 designs with 100 or more mutations, used to model the experimental noise. Two sided Kolmogorov-Smirnov (K-S) test $p = 0.943$, indicating no significant deviation from normality. The vertical line corresponds to the noise threshold of $p = 0.001$.

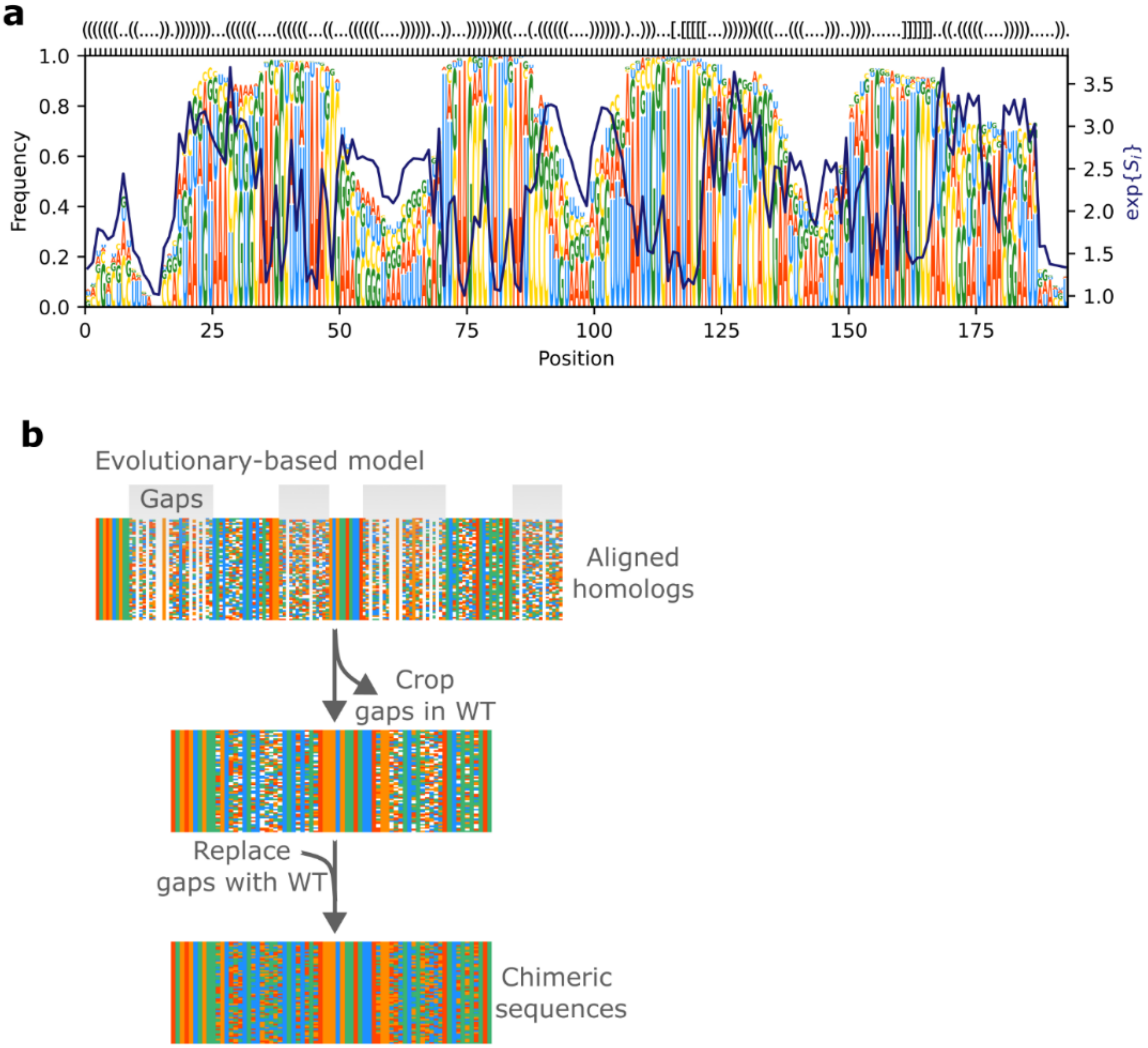

Supplementary Figure 5. Construction of the Multiple Sequence Alignment (MSA). a, Natural diversity by position in the MSA. Top row: Secondary structure of Azo in dot bracket notation. Main panel: Logo representation indicating the frequency of nucleotides per position. The solid curve represents the effective number of nucleotides per position (exponential of Shannon entropy). **b,** Design of the chimera sequences. Homologs of Azo were found and aligned on the wild type sequence and secondary structure using the Infernal package. Insertions with respect to the wild type sequence were removed such that all sequences were all the same length (193 nucleotides). The deletions were then replaced by the wild type nucleotide at that position.

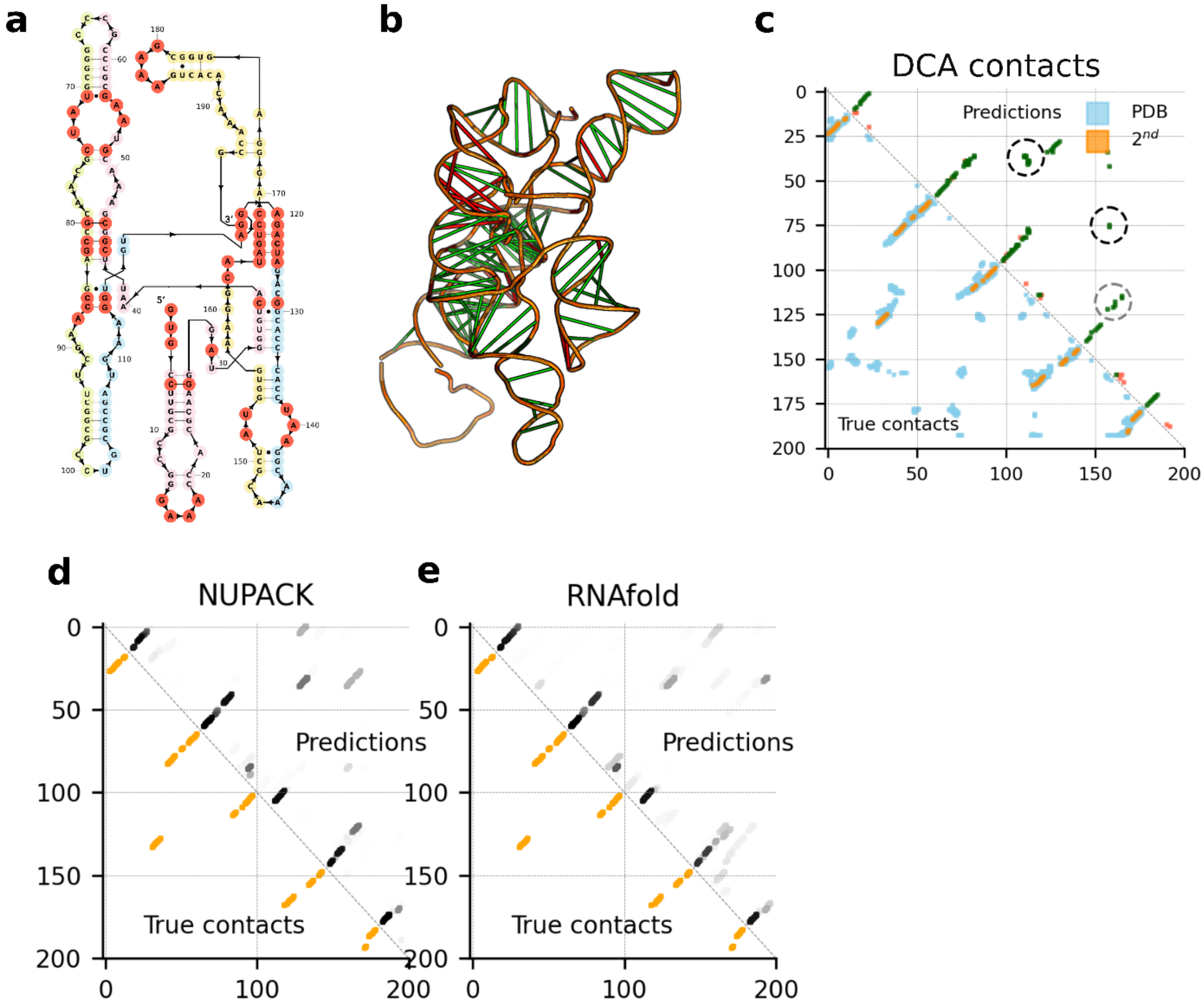

Supplementary Figure 6. Structure prediction using evolutionary conservation and biophysical models. a, Tertiary constraints used with the BPR and SB models. Red positions correspond to positions that are in contact in the tertiary structure[9]. These positions were held fixed in the wild type nucleotide when applying the 3D constraint design. **b,** Tertiary structure of Azoarcus X-ray structure 1G9B. We show the correct (green) and incorrect (red) predictions of contacts using DCA. **c,** The contact map inferred with DCA compared with the true tertiary and secondary contact also displays the predicted tertiary contacts (circled in gray). **d-e,** Base pair probability matrices predicted with secondary structure algorithms: **d,** with NUPACK (which account explicitly for pseudoknot) and **e,** with RNAfold (which do not account for pseudoknot).

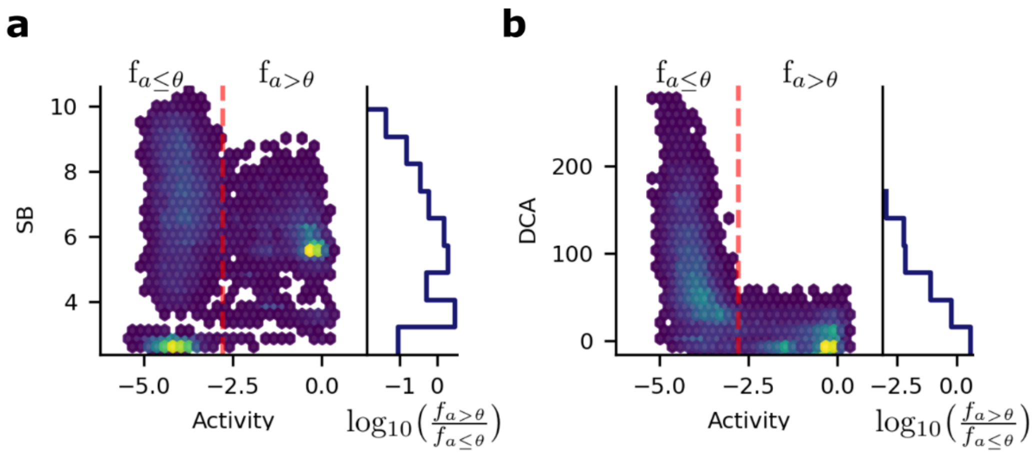

Supplementary Figure 7. Structure-based (SB) score and evolutionary (DCA) score compared to the experimental activity. a, the graph shows the distribution of SB against the measured activity for the tested sequences where the color gradient represents the density of designs. The red dashed line represents the p-value=10^{-3} threshold Θ=-2.76. The curve on the right displays the ratio between designs below and above the threshold. **b,** Same but for the DCA score.

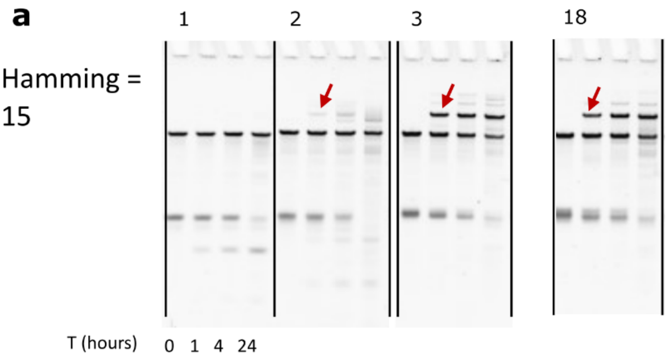

Column	Sequence Name	Step 2	Step 1+2
1	dca.T1.15.2	-0.431	-2.494
2	dca.T1.15.3	-0.156	-1.059
3	dca.T1.15.4	0.511	0.479
4	dca.T1.30.3	-0.713	-2.498
5	dca.T1.30.5	-0.375	-0.397
6	dca.T1.30.4	0.215	-1.499
7	dca.T1.45.3	-0.788	-2.168
8	dca.T1.45.6	-0.599	-1.365
9	dca.T1.45.4	-0.075	-1.168
10	dca.T1.45.7	-0.382	-0.602
11	dca.T1.45.5	-0.321	-0.357
12	dca.T1.50.2	-1.110	-1.654
13	dca.T1.50.1	-0.612	-1.293
14	dca.T1.55.2	-1.352	-2.703
15	dca.T1.55.1	-0.358	-1.522
16	dca.T1.60.2	0.007	-1.330
17	dca.T1.60.1	0.570	-1.232
18	Azo	0	0

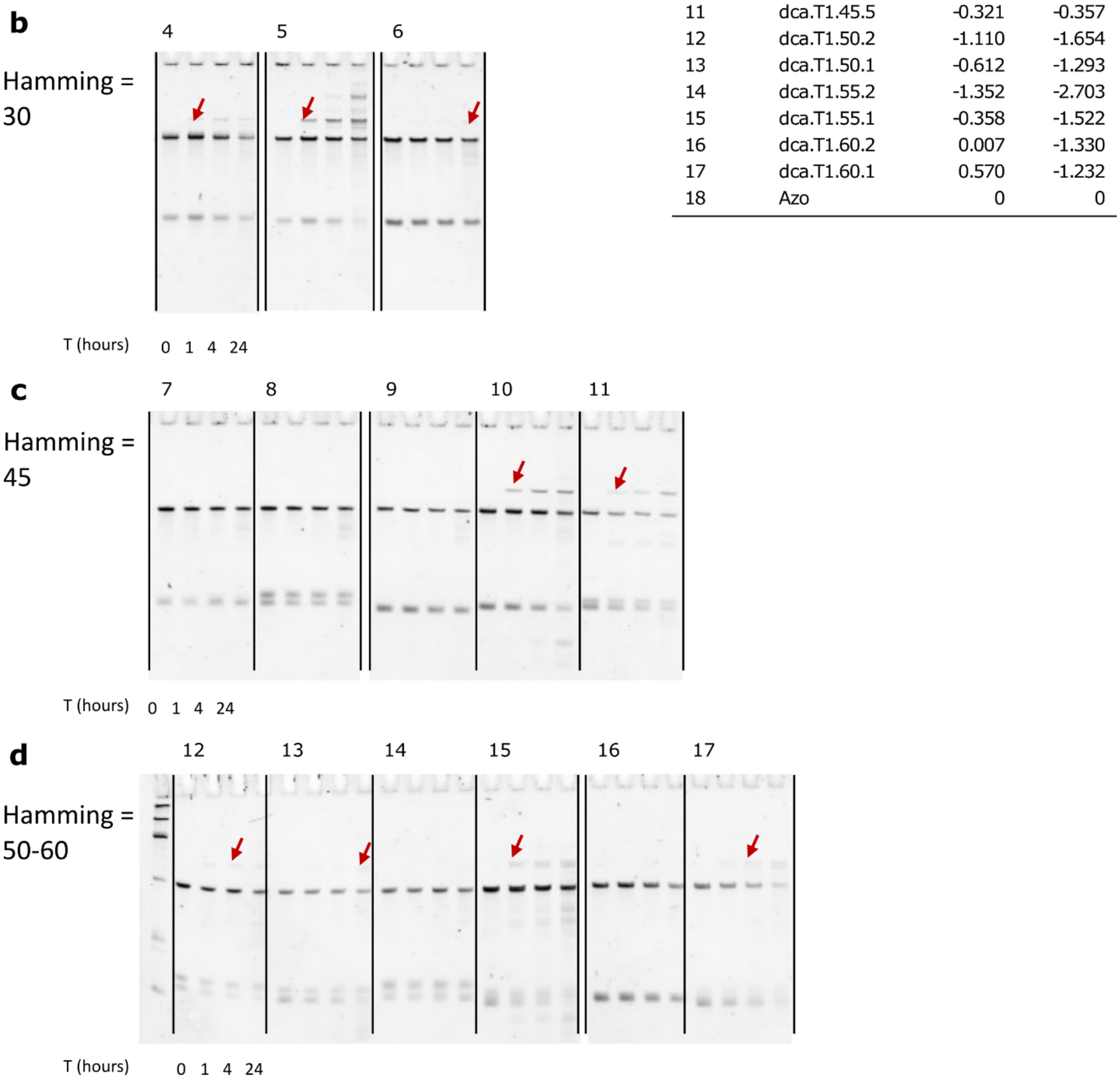

Supplementary Figure 8. Individual two-fragment self-reproduction assay for DCA designs. Self-reproduction assay for 17 DCA designs. All designs tested were considered active (with an activity score above the activity threshold of -2.76). The table indicates the design name and their activity scores (step 2 refers to the ability of the design to take the substrate and step 1+2 refers to the ability of the design to remove the exon and then take the substrate). The red arrow shows the presence of the recombined covalent ribozyme.

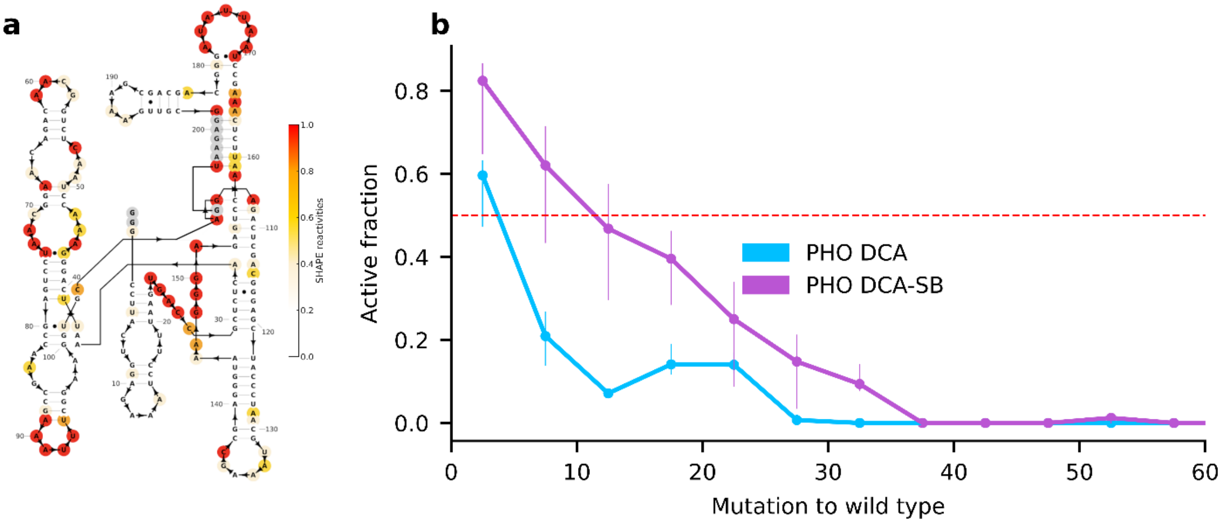

Supplementary Figure 9. Self-splicing design and experimental activity measured for Phormidium variants. a, Shape reactivities projected on the 2D structure of Phordium group I intron. **b,** The top panel shows the activity (relative to Azoarcus activity) across the number of mutations from the wild type Phormidium sequence. Dots are the active fraction. Error bars upper (lower) bound of vertical bars is the active fraction including (excluding) activity scores within the 98,5 percentile of the measurement error distribution around the threshold. For DCA, N=1336 designs were measured whereas for DCA-SB N=1332 designs were measured.

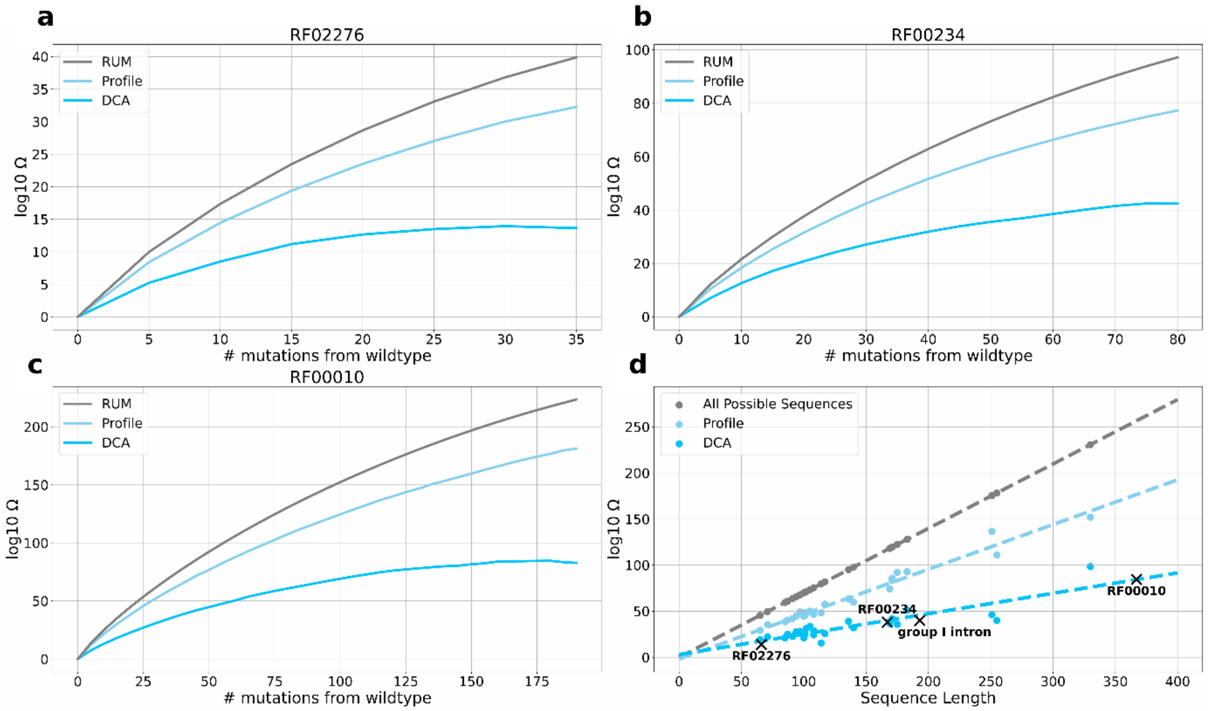

Supplementary Figure 10. Diversification potential of ribozymes. a) Curve of accessible diversity (support size) as a function of the number of mutations introduced in an initial sequence of the RF02276 RNA family of Hammerhead ribozymes (458 members, 66 nucleotides) for Random Uniform Mutagenesis, the Profile model (position-wise nucleotide frequencies), and the DCA model. Each row represents the frequency of mutations in all four nucleotides where dots represent the wild type nucleotides. **b**) Same as panel (a) for the RF00234 RNA family of glmS riboswitch ribozymes (943 members, 167 nucleotides). **c**) Same as panel (a) for the RF00010 RNA family of Bacterial RNase P class A (8929 members, average length 367 nucleotides). **d**) Scaling laws of diversity as a function of length, where the DCA support size estimate is shown for the families of panels (a-c).

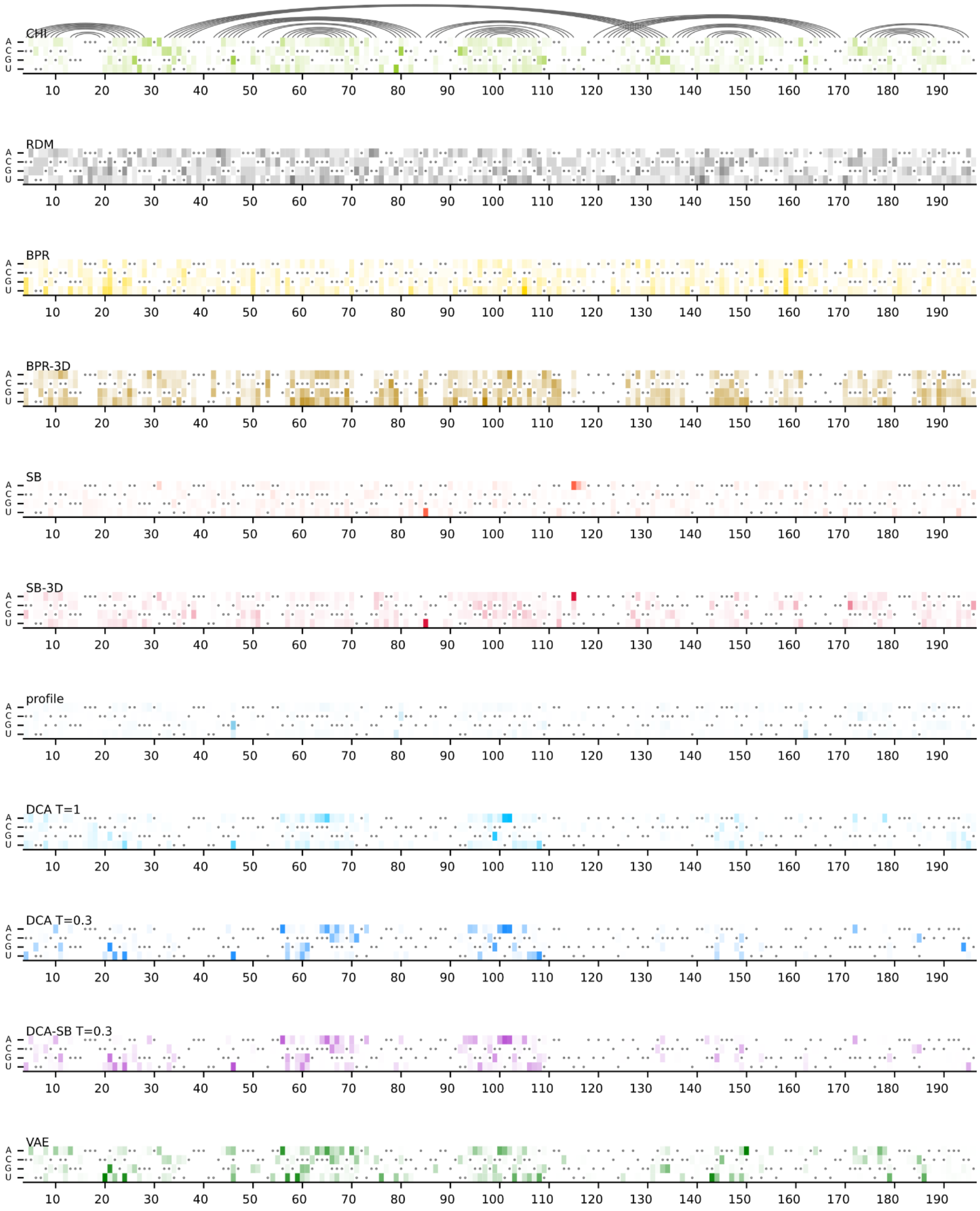

Supplementary Figure 11. Distribution of mutations across the positions among active sequences per model. Each row represents the frequency of mutations in all four nucleotides where dots represent the wild type nucleotides.

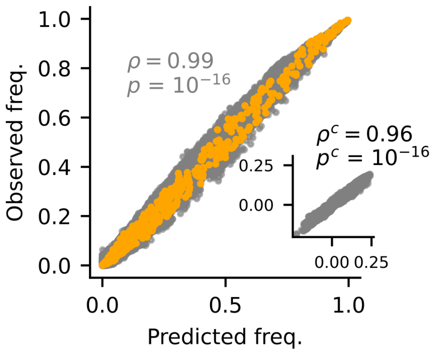

Supplementary Figure 12. Reproduction of first- and second-order statistics with the DCA model. The main scatter plot shows the correlation of single (orange) and pairwise (gray) frequencies between the MSA and the DCA predictions. The Pearson correlation ρ between observed and predicted frequencies is 0.99 with a two-sided test, p=10^{-16}, which is the numerical precision. For the connected correlations $c_{ij}(a,b) = f_{ij}(a,b) - f_i(a) \times f_j(b)$ shown in the inserted scatter plot, the Pearson correlation $\rho^c = 0.96$ with a two-sided p=10^{-16} (numerical precision).

Algorithm 1: *bl-dca* implementation

```
Data: MSA, N, K Result: DCA
// Initialize the DCA parameters to zeros
DCA ← 0;
// Compute f_i and f_ij from the MSA
F ← frequencies(MSA);
for i ∈ 1 → N do
    // Pick a random sequence from the MSA
    s ← pick_one(MSA);
    // Perform K Monte Carlo steps to mutate the sequence s
    for k ∈ 1 → K do
        // Propose a mutation at a random position
        mut← mutate(s)
        // Apply the Metropolis criterion, and update s if
          accepted
        if Metropolis(mut) then s ← mut;
    // Update the DCA parameters
    for i ∈ 1 → L do
        // Update h_i
        for n_i ∈ {A, C, G, U, '−'} do
            DCA[i,n_i] -= (F[i,n_i] - δn_i, s_i) ×η;
        // Update J_ij
        for j ∈ i → L do
            for n_i ∈ {A, C, G, U, '−'} do
                for n_j ∈ {A, C, G, U, '−'} do
                    DCA[i,j,n_i,n_j] -= (F[i,j,n_i,n_j] - δn_i, s_i ×δn_j, s_j) ×η;
    // k is choosen such that η converges to 10^−4 in the end
    η ← η × k;
```

Supplementary Figure 13. Pseudo-code of the BL-DCA implementation derived from (*7*). The algorithm takes as argument the MSA, the number of parameter update steps N, and the number of MCMC steps. The learning parameter $\boldsymbol{\eta}$ is typically set to 0.001.

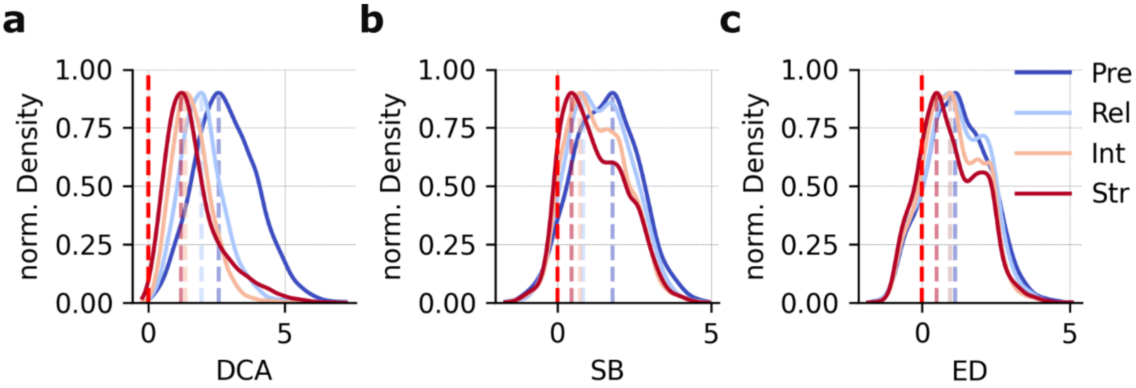

Supplementary Figure 14. Validation of models on the benchmark mutational dataset. Cross validation of the DCA, structure-based (SB), ensemble defect (ED) scores on an independent experimental dataset. Four experimental conditions were tested from the less stringent to the most (respectively in this order Pre, Rel, Int, and Str). The distributions represent the scores computed on the set of sequences obtained with that selection pressure, which we normalized such that the maximum (the mode) of the distribution is 0.9. The dashed lines show the position of the mode with respect to the wild type (defined at 0).

name	# designs	# detected	# active designs	L_{50}	fraction at L_{50}	p at L_{50}	L_{max}	fraction at L_{max}	p L_{max}
**RUM**	1687	1678	188	5	133/139 (95.7%)	< 1.00e-300	10	51/170 (30%)	7.47e-110
**BPR**	1350	1344	156	15	41/69 (59.4%)	< 1.63e-104	20	4/76 (5.3%)	1.21e-6
**BPR+3D**	1350	1348	231	15	32/59 (54.2%)	4.72e-80	35	3/70 (4.3%)	5.21e-5
**SB**	4200	4156	274	10	241/278 (86.7%)	< 1.00e-300	20	13/230 (5.7%)	4.68e-19
**SB-3D**	1350	1347	231	10	129/147 (87.8)%	< 1.00e-300	40	3/99 (3.0%)	1.46e-4
**PRO**	1800	1799	197	5	188/200 (94.0%)	< 1.00e-300	15	7/200 (3.5%)	1.93e-9
**DCA T=1**	2547	2513	772	20	118/150 (78.7%)	< 1.00e-300	60	3/149 (2.0%)	4.84e-4
**DCA T=0.3**	2203	2194	1344	45	66/104 (63.5%)	3.48e-170	60	6/188 (3.2%)	4.84e-8
**DCA-SB**	4200	4190	1634	55	150/175 (85.7%)	< 1.00e-300	65	8/116 (6.9%)	5.77e-13
**VAE**	2800	2773	818	15	253/280 (90.4%)	< 1.00e-300	60	6/269 (2.2%)	3.97e-7
**CHI**	733	729	46	//	//	//	65	5/96 (5.2%)	5.67e-8
**Total**	24220	24071	5891						

Supplementary Table S1. Comparison of models. Model acronym; Number of designs generated; Number of designs detected after sequencing; Number of designs with an activity score larger than the threshold; Highest mutation number of the L_{50} bin; Number of active designs over total number in the L_{50} bin; p-value from the one-sided Binomial test for 50% activity in the L_{50} bin (see Methods); Highest mutation number of the L_{max} bin ; Number of active designs over total number in the L_{max} bin ; p-value from a one-sided Binomial test for 0.1% activity in the L_{max} bin (see Methods).

distance → model ↓	5	10	15	20	25	30	35	40	45	50	55	60	65	70	75	80
**RUM**	11.71	20.96	29.08	36.42	43.17	49.42	55.25	60.71	65.82	70.62	75.13	79.37	83.35	87.07	90.55	93.80
**PRO**	9.48	16.90	23.39	29.14	34.46	39.28	43.70	47.63	51.56	55.01	58.25	61.20	63.96	66.44	68.65	70.82
**BPR**	10.17	17.91	24.55	30.45	35.79	40.68	45.20	49.38	53.27	56.90	60.30	63.47	66.44	69.20	71.78	74.18
**BPR-3D**	9.22	16.00	21.68	26.62	31.01	34.95	38.50	41.72	44.63	47.26	49.63	51.75	53.63	55.26	56.66	57.81
**DCA T=1**	7.04	12.58	17.28	21.41	24.94	28.11	30.83	33.37	35.57	37.52	39.29	40.62	41.87	43.03	43.96	44.36
**DCA T=0.3**	1.74	2.86	4.55	6.53	8.20	8.63	8.10									
**DCA-SB**	5.71	9.72	13.9													

Supplementary Table S2. Computed model support sizes. Values are in $\log_{10}$ and are the theoretical values before correction by the experimental active fraction. Highlighted in bold is the support size corresponding to L_{max}. For the DCA-SB model, the support size is reported using the least-constrained parameter choice (alpha = 0.4). For the models DCA at T=0.3 and DCA-SB, support size estimation becomes infeasible in practice beyond a certain distance due to ergodicity issues. DCA T=1 support sizes have been reduced by a factor of ten because we only experimentally tested the sequences in the top 10% quantile of the DCA score.

Sequence name	sequence	Distance from Azo
**Azo**	GUGCCUUGCGCCGGGAAACCACGCAAGGGAUGGUGUCAAAUUCGGCGA AACCUAAGCGCCCGCCCGGGCGUAUGGCAACGCCGAGCCAAGCUUCGGC GCCUGCGCCGAUGAAGGUGUAGAGACUAGACGGCACCCACCUAAGGCAA ACGCUAUGGUGAAGGCAUAGUCCAGGGAGUGGCGAAAGUCACACAAAC CGG	0
**dca_sb_1434_60_70**	GUGCACUGCUCCGGGAAACCAAGUAGUGAAUGCUCUCAAAUUCAGGGA AACCUAAAUCUGGUAGUCCAGAUAAGGCAACCCUGAGCCAAGCCAAGUC ACCUAUGACUUGGAAGGUGCAGAGACUCGACGGGAGCUACCUAACGGU UAGCCGAGGGUAAAGGGAGAGUCCAAUUACUGACGAAAGUCAGACAAA GAGG	65
**chimeric_466**	GUGGCUUGCGCCGGGAAACCACGCAAGCAAAUCAGGCUAAUUCGGGGA ACGCCUAACGCCCGCCCGGGCGUAGGUCAAUCCCGAGCUAAGUCCCCGA UUUAAUUGGGGUAAAUGUGUAGAGACUAUAUACCUGACACCAGUGGCA AACGCUAUGGUGAUGAGAUAGUCCAGUCCUUUUGGUAACAAGAGGAAA CCGG	65
**chimeric_184**	GUGCCAUGCGCCGGGAAACCACGCAUGAUAACUUGGCUAAUUCGGGGA AGUCCAAGCGCCCGCCCGGGCGUAUGAUAAUCCCGAGCUAAAUUCUCCG CCAUGGCGGGGUAAAUGUGUAGAGACUAUAUACCAGGGACCUAAAGCA AACGCCAAGGUCAUAAGAUAGUCCAAACCUUUAAGUAAUUAGAGGAAA CCGG	64
**dca_f_mutations_213**	GUGCAUUCUGUUAGGAAACUUUAGAAUGAAUGGUGUCAAAUUCGGUGA AACCUAAGUCUUUGCCAAAAGAUAAGGCAACGCCGAGCCAAGCUCAUUU AGAAACAAUUGAGAAGGUGUAACGACUAGACGGCACCCACCUAAGGUA AUCACAAUGGUGAAGGCAUAGUCUAGAGAGCAACCAAAGUUGCACAGG CACG	60

**dca_sb_1469_60_70**	GUGUAUGCCUGCGGGAAACCUAGGCAUGAAUGUGGUCAAAUUCGAUGAAACCUAAAUAGUGACAACACUAUAAGGCAAUAUCGAGCCAAGCCUUAUCAGUAAUGAUAAGGAAGGUGUAGAGACUAGACGGCCACCACCUAAAGGAAACCCUACGGUGAAGGUAUAGUCCAGAGAGUGGGAAACUCACACGAACUGG	60
**dca_sb_1496_60_70**	GUGCAUUCAGGCAGGAAACUUCUGAGUGAAUGUGCUCAAAUUCGGUGAAACCUAAAGAGUGGAAACACUCUAAGGGAAUACCGAGCCAAGCCUUUUCAUCAAUGAAAAGGAAGGUGUAGAGACUAGACGGGCACCACCUAAAGGAAAACCUAUGGUGAAGGUAUAGUCCAGAGAGUGGGAAACUCACACAUACUGG	60
**chimeric_382**	GUGCCUUCCGCCGGGAAACCACGGAAGAUUACUUGCCAAAUUCGGGGAAGCCCACGUUCCCGCCCGGGAACUAGGUAAUCCCGAGCUAAGCUCUGAUGUUUGUAUCAGAGAAAGUGUAGAGACUAGAUGGUAAGCACCUUAAAUAAUGAUUUAGGUGAAGGGAUAGUCCAGACUACAGCGAAAGUUGUAGAAACCGG	62
**dca_f_mutations_238**	GUGCUCUAAUAAGGGAAACCGAUUAGUGAAUGUGGUCAAAUUCAGGGAAGCUUAAGGAUAUUAUUAUAUCUAUGGUAACCCUGAGCCAAGCUUAGAAGCAAAUUUCUUUGAAGGUGCAGAGACUAGACGGCCACCACCUAAGGGAAACCCUAGGGUGAAGGGAUAGUCCAGGGAGUGACGAAAGUCACACGAAUUCG	60

Supplementary Table S3. Active sequences at maximum distances. Azo is the reference sequence. In this table, we only consider sequences that belong to bins where the active fraction is established with $p<0.001$. Sequences 'dca_sb_1434_60_70' and 'chimeric_466' are the ones found active at 65 mutations from Azo and are 99 mutations away from each other. The other pairs of sequences 99 mutations away from each other are: 'chimeric_466' and 'dca_f_mutations_213', 'chimeric_184' and 'dca_f_mutations_213', 'dca_sb_1469_60_70' and 'chimeric_184', 'dca_sb_1496_60_70' and 'chimeric_184'. The CHI 'chimeric_382' sequence is 59 mutations away from any other CHI sequence. The 'dca_f_mutations_238' generated by DCA at T=1 is 55 mutations away from any CHI sequence.

Pool	[MgCl2] (mM)	Avg. length (nuc)	Nb. seq.
**Pre**	-	173.9	12740
**Rel**	25	174.5	3346
**Int**	10	174.6	25586
**Str**	2	174.7	11803

Supplementary Table S4. Benchmark mutational dataset. Size and sequence length of the four pools of sequences used for the DCA benchmarking with their average DCA score (relative to Azo), extracted from[10].

Supplementary References

1. Calvanese, F., Lambert, C. N., Nghe, P., Zamponi, F. & Weigt, M. Towards parsimonious generative modeling of RNA families. *Nucleic Acids Research* **52**, 5465–5477 (2024).
2. Cuturello, F., Tiana, G. & Bussi, G. Assessing the accuracy of direct-coupling analysis for RNA contact prediction. *RNA* **26**, 637–647 (2020).
3. Ding, X., Zou, Z. & Brooks Iii, C. L. Deciphering protein evolution and fitness landscapes with latent space models. *Nat Commun* **10**, 5644 (2019).
4. Mathews, D. H. Incorporating chemical modification constraints into a dynamic programming algorithm for prediction of RNA secondary structure. in *Proceedings of the National Academy of Sciences* vol. 101 7287–7292 (2004).
5. Zuker, M. & Stiegler, P. Optimal computer folding of large RNA sequences using thermodynamics and auxiliary information. *Nucleic Acids Res* **9**, 133–148 (1981).
6. McCaskill, J. S. The equilibrium partition function and base pair binding probabilities for RNA secondary structure. *Biopolymers: Original Research on Biomolecules* **29**, 1105–1119 (1990).
7. Lorenz, R. *et al.* ViennaRNA Package 2.0. *Algorithms Mol Biol* **6**, 26 (2011).
8. Zadeh, J. N. *et al.* NUPACK: Analysis and design of nucleic acid systems. *J Comput Chem* **32**, 170–173 (2011).
9. Mustoe, A. M., Al-Hashimi, H. M. & Brooks, C. L. Secondary structure encodes a cooperative tertiary folding funnel in the Azoarcus ribozyme. *Nucleic Acids Research* **44**, 402–412 (2016).

10. Hayden, E. J., Bendixsen, D. P. & Wagner, A. Intramolecular phenotypic capacitance in a modular RNA molecule. *Proceedings of the National Academy of Sciences* **112**, 12444–12449 (2015).

11. Dirks, R. M. & Pierce, N. A. An algorithm for computing nucleic acid base-pairing probabilities including pseudoknots. *J Comput Chem* **25**, 1295–1304 (2004).

12. Cover, T. M. & Thomas, J. A. *Elements of Information Theory*. (Wiley, New York, 1999).

13. Kardar, M. *Statistical Physics of Fields*. (Cambridge University Press, 2007). doi:10.1017/CBO9780511815881.
